# Antitumor Activity of a 5-Hydroxy-1*H*-Pyrrol-2-(5*H*)-One-Based Synthetic Small Molecule *In Vitro* and *In Vivo*

**DOI:** 10.1371/journal.pone.0128928

**Published:** 2015-06-04

**Authors:** Yunyun Geng, Xiaojian Wang, Luo Yang, Haili Sun, Yongqiang Wang, Yue Zhao, Ruiping She, Mei-Xiang Wang, De-Xian Wang, Jun Tang

**Affiliations:** 1 State Key Laboratory of Agrobiotechnology and College of Veterinary Medicine, China Agricultural University, Beijing, 100193, China; 2 Beijing National Laboratory for Molecular Sciences, CAS Key Laboratory of Molecular Recognition and Function, Institute of Chemistry, Chinese Academy of Sciences, Beijing, 100190, China; 3 The Key Laboratory of Bioorganic Phosphorus Chemistry & Chemical Biology (Ministry of Education), Department of Chemistry, Tsinghua University, Beijing, 100084, China; Peking University Health Science Center, CHINA

## Abstract

Alternative chemo-reagents are in great demand because chemotherapy resistance is one of the major challenges in current cancer treatment. 5-hydoxy-1*H*-pyrrol-2-(5*H*)-one is an important N-heterocyclic scaffold that is present in natural products and medicinal chemistry. However, its antitumor activity has not been systematically explored. In this study, we screened a panel of 5-hydoxy-1*H*-pyrrol-2-(5*H*)-one derivatives and identified compound **1d** as possessing strong anti-proliferative activity in multiple cancer cell lines. Cell cycle analysis revealed that **1d** can induce S-phase cell cycle arrest and that HCT116 was sensitive to **1d**-induced apoptosis. Further analysis indicated that **1d** preferentially induced DNA damage and p53 activation in HCT116 cells and that **1d**-induced apoptosis is partly dependent on p53. Furthermore, we showed that **1d** significantly suppressed tumor growth in xenograft tumor models *in vivo*. Taken together, our results suggest that 5-hydoxy-1*H*-pyrrol-2-(5*H*)-one derivatives bear potential antitumor activity and that **1d** is an effective agent for cancer treatment.

## Introduction

Cancer is one of the most prevalent diseases worldwide and is closely associated with mortality [1]. For patients with advanced tumors, chemotherapy is often employed. However, the conventional agents face the issue of drug resistance [2, 3]. Thus, searching for alternative cytotoxic agents with different mechanisms of action may provide a solution to reduce drug resistance and achieve better outcomes.

A good source for alternative cytotoxic agents is natural products and their derivatives, which account for nearly half of the drugs that are currently used in cancer therapy [4]. 5-hydoxy-1*H*-pyrrol-2-(5*H*)-one is an important N-heterocyclic scaffold in both natural products and medicinal chemistry. Many natural products bearing this heterocyclic structure exhibit important biological activities. For example, the fungi metabolite oteromycin [5] is an effective antagonist of endothelin receptor and can inhibit the integrase activity of human immunodeficiency virus. Epolactaene, a microbial metabolite isolated from Penicillium sp., contains an analog of 5-hydoxy-1*H*-pyrrol-2-(5*H*)-one. This metabolite possesses potential antitumor activities, such as cell cycle arrest and neurite outgrowth stimulation, in human neuroblastoma SH-SY5Y cells [6] and induces apoptosis in the human leukemia B-cell line BALL-1 [7]. A recent study with synthetic derivatives of epolactaene demonstrated that the growth inhibitory effect of the compound on human cancer cells is mostly induced by the N-heterocyclic moiety [8]. Although 5-hydoxy-1*H*-pyrrol-2-(5*H*)-one derivatives have potential antitumor activity, this activity has rarely been explored because their synthetic difficulty makes the unavailable. Recently, a highly efficient and expedient protocol for synthesis of 5-hydoxy-1*H*-pyrrol-2-(5*H*)-ones was developed [9], and this protocol permits the synthesis of a variety of this compound’s derivatives for the study of their antitumor activity.

In this study, we examined the effect of a series of derivatives of 5-hydoxy-1*H*-pyrrol-2-(5*H*)-one on the growth of cancer cells and identified **1d** as a potent cell growth inhibitor. We found that **1d** induced cell cycle arrest during S-phase and that HCT116 is sensitive to **1d**-induced apoptosis. The anti-tumor activity of **1d** was confirmed *in vivo* using two xenograft tumor models. Our data implicate **1d** as an efficient anti-tumor agent for therapeutic use.

## Materials and Methods

### Cell lines

Human colorectal carcinoma cell line HCT116, cervix adenocarcinoma cell line HeLa, osteosarcoma cell line U2OS, non-small cell lung cancer cell line H1299, liver hepatocellular carcinoma cell line HepG2 and normal human lung fibroblasts cell line IMR90 were obtained from the American Type Culture Collection (ATCC). Gastric adenocarinoma AGS cells and colorectal carcinoma HT29 cells were originally purchased from the American Type Culture Collection (ATCC); we obtained these two cell lines from Dr. Wenhai Feng (China Agricultural University) and Dr. Like Qu (Peking University School of Oncology), respectively. HCT116-p53^-/-^ cells were originally obtained from Dr. Bert Vogelstein (Johns Hopkins University) [10]. HCT116, HT29, HeLa, U2OS, H1299, HepG2 and IMR90 cells were maintained in Dulbecco's modified Eagle's medium (DMEM), whereas AGS cells were maintained in Ham's F12 medium. Both media were supplemented with 10% fetal bovine serum (FBS), 100 U/ml penicillin and 100 U/ml streptomycin. All cells were cultured at 37°C in a humidified incubator supplied with 5% CO_2_.

### Reagents

Derivatives of 5-hydoxy-1*H*-pyrrol-2-(5*H*)-one were synthesized by the authors according to our previously reported protocol [9] and dissolved in DMSO to make stocks. Doxorubicin (Dox) was purchased from Sigma-Aldrich. Antibodies included the following: p53 (DO-1), Caspase-3 and β-actin (all from Santa Cruz Biotechnology); human Mdm2 (Ab-1, Calbiochem); p21 (BD Pharmingen); phospho—H2A.X (Ser139, 05–636, Millipore); and phospho-p53 (Ser15), phospho-p53 (Ser46) and phospho-Chk1 (Ser345, 133D3) (all from Cell Signaling).

### Western blotting

Western blotting was performed as described previously [11]. Briefly, whole cell lysates were prepared in lysis buffer. Protein samples were resolved via SDS–PAGE and transferred onto a PVDF membrane, which was blocked in 5% skim milk and probed with the indicated antibodies.

### Immunofluorescence staining

The cells were seeded on coverslips and cultured overnight. After treatment with **1d** (5 μg/ml), Dox (0.4 μg/ml) or DMSO (control) for 8 hours, the cells were fixed in 4% paraformaldehyde for 15 minutes and then permeabilized in 0.2% Triton X-100 on ice. The cells were blocked with 1% BSA and incubated with the γ-H2A.X antibody, followed by a FITC-conjugated secondary antibody. Nuclei were counterstained with 4’,6’-diamidino-2-phenylindole (DAPI; Beyotime). Images were acquired with a Leica Wetzlar GmbH microscope.

### Cell survival analysis

For crystal violet staining, 5×10^3^−1×10^4^ of cells were plated in 24-well plates and grown overnight. The cells were then given fresh medium containing 1 μg/ml of **1d** or vehicle and grown for another 1 to 6 days. The medium containing **1d** or vehicle was replaced every two days if needed. The cells were washed with PBS and stained daily with crystal violet.

### MTT assay

Approximately 1×10^3^−1×10^4^ cells were seeded in 96-well plates and cultured overnight. The cells were then treated with different concentrations of compounds and incubated for 48 hours. MTT was added, and the cells were incubated at 37°C for 4 hours, followed by aspiration of the medium and addition of 200 μl of DMSO. Absorbance was measured at a wavelength of 490 nm using a microplate reader (Multiskan GO, Thermo Fisher Scientific).

### Flow cytometry analysis

Cells were exposed to different concentrations of **1d** or DMSO for 24 hours and then harvested by pooling the floating and attached cells. After two washes with cold PBS, the cells were fixed with 70% ethanol on ice for 2 hours, washed with cold PBS and then suspended in 300 μl of propidium iodide (PI; Sigma) staining solution (PI 100 μg/ml, RNaseA 100 μg/ml, Triton X-100 0.1%, PBS pH 7.4). The samples were analyzed via flow cytometry using a FACSCalibur flow cytometer (Becton Dickinson). For the cell apoptosis analysis, cells were treated and collected as described above and then subjected to Annexin V and PI staining using an Annexin V-FITC Apoptosis Detection Kit (Biosea), according to the manufacturer’s protocol. Apoptotic cells were then analyzed using flow cytometry.

### Real-time RT-PCR

Total RNA was isolated as described previously [11]. Briefly, total RNA was isolated using TRIzol (Invitrogen), and an equal amount (4 μg) of RNA from each sample was reverse transcribed to cDNA using the First Strand cDNA Synthesis Kit (TAKARA) according to the manufacturer's instructions. The same amounts of cDNA products were amplified using quantitative real-time PCR (qRT-PCR) using SYBR Premix Ex Taq II (TAKARA) and a 7500 Fast Real-Time PCR System (Applied Biosystems). The following primers were used: p21: 5’: GCGTTCACAGGTGTTTCT-3’ and 3'-GTCCACTGGGCCGAAGAG-5’[12]; The primers for PUMA [13], Mdm2 [12] and β–actin [14]were described previously.

### Xenograft tumors

Six-week-old female athymic BALB/c nu/nu mice were obtained from Vital River Laboratories (VRL). Animal care and protocols were approved by the Animal Welfare Committee of China Agricultural University and followed the NIH Guidelines on the Use of Laboratory Animals. All efforts were made to minimize suffering. HCT116 or H1299 cells (6 ×10^6^ cells in 100 μl) were injected subcutaneously into the left flank of athymic BALB/c nu/nu mice to initiate tumor growth. After 4 days, when tumors were palpable, mice were randomized (n = 6 to 8 per group) and administered with **1d** or DMSO intraperitoneally (25 mg/kg) daily for the first week and every two days from day 8 to 18. The tumor volume was measured and calculated by the formula 0.5236 ab^2^, wherein a represents the long axis and b represents the short axis of the tumor. Body weight and average diet consumption were measured every three days. At the end of the experiments, the mice were sacrificed under ether anesthesia by cervical dislocation. Tumors were excised and weighed.

### Statistical analysis

Data are presented as the mean ± SEM, and the *P* values were determined using two-tailed Student^’^s *t* test. GraphPad Prism 5.0 (GraphPad Software, Inc.) was used for the analyses. *P* < 0.05 was considered statistically significant.

## Results

### Derivatives of 5-hydoxy-1*H*-pyrrol-2-(5*H*)-one possess cell growth inhibitory activity

To explore whether 5-hydoxy-1*H*-pyrrol-2-(5*H*)-ones have any anti-tumor activity, we synthesized a series of derivatives, as indicated in S1 Fig, and then examined the effect of each compound on the growth of HCT116 cells using MTT assay. Treating cells with these compounds for 48 hr significantly inhibited cell growth (S1 Table), even though the growth inhibitory activity of these compounds is not as strong as that of the commonly used therapeutic drug Doxorubicin (Dox). Among these compounds, compound **1d** showed the highest inhibition. We therefore chose **1d** for further study.

### Compound 1d inhibits the growth of multiple cancer cells

**Fig 1:**
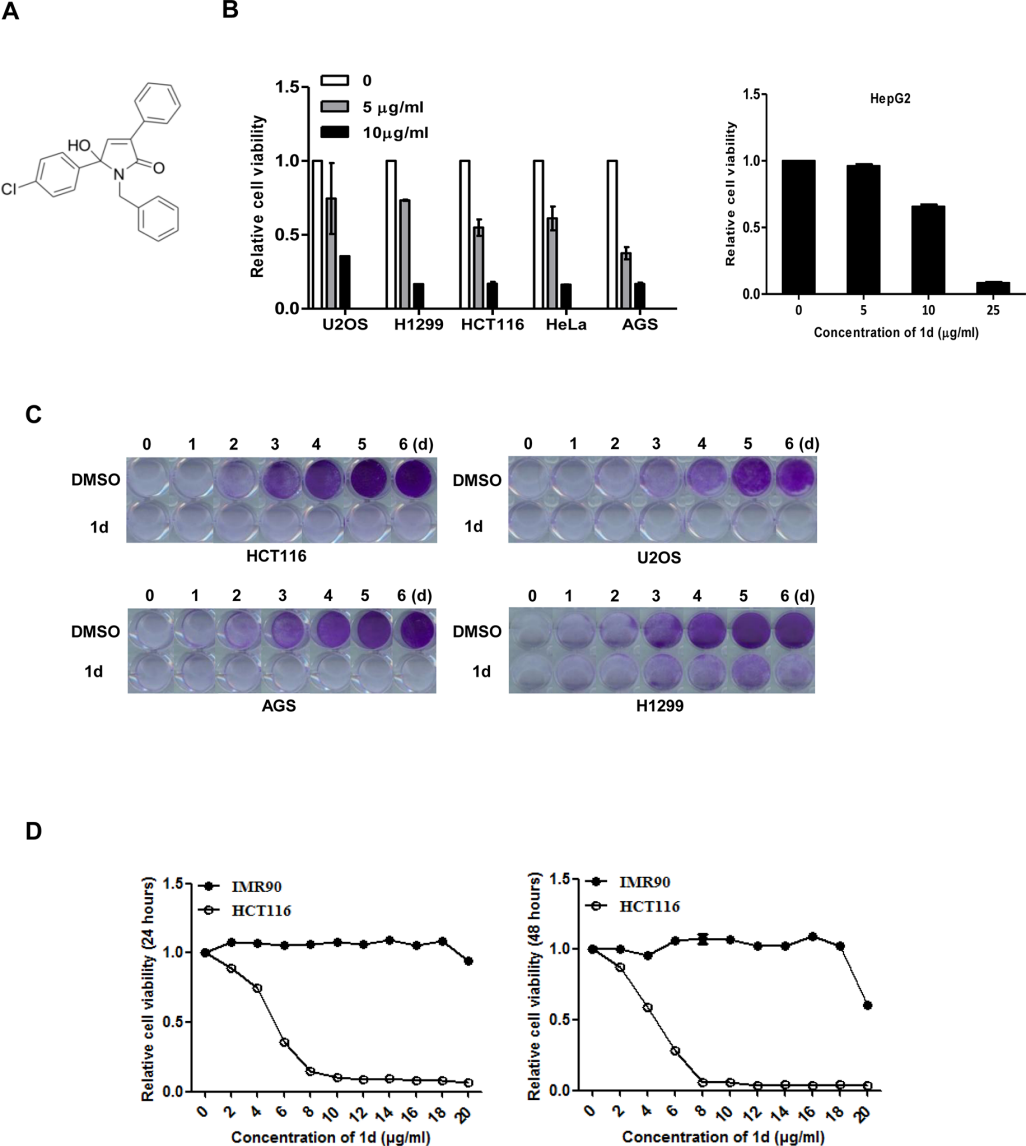
1d suppresses the growth of multiple cancer cell lines. (A) The chemical structure of **1d**. (B) Cells were treated with **1d** at 5 or 10 μg/ml or with DMSO (control) for 48 h and were analyzed using the MTT assay. Data are shown as the means of 2 independent experiments ± SEM. (C) The indicated cells were treated with **1d** (1 μg/ml) or DMSO for the indicated days and stained with crystal violet. (D) HCT116 and IMR90 cells were treated with a serial diluted **1d** for 24 h (left) and 48 h (right) and were analyzed using the MTT assay.

**1d** is a small chemical compound with a MW of 375, and its structure shown in Fig 1A. We confirmed the cell growth inhibitory effect of **1d** on multiple cell lines. The MTT assay indicated that **1d** was more potent to AGS, HCT116, U2OS, H1299 and HeLa cells than to HepG2 cells (Fig 1B). The long-term culture of **1d**-sensitive cell lines in the presence of **1d** at a concentration of 1 μg/ml significantly suppressed cell growth (Fig 1C). We also assessed the effect of **1d** on normal cell growth. Treating HCT116 and the normal human lung fibroblast IMR90 cells with the same serial diluted **1d** revealed that IMR90 cells are much less sensitive to **1d**-induced cell growth inhibition than HCT116 cells (Fig 1D). Moreover, **1d** at a concentration of 10 μg/ml had no effect on IMR90 growth, but significantly suppressed the growth of a wide range of cancer cell lines (Fig 1B and 1D), indicating **1d** is less effective in inhibiting normal cell growth than that of cancer cells. Altogether, these data suggest that **1d** has potent anti-tumor activity.

### Compound 1d induces S-phase cell cycle arrest in multiple cell lines

To understand the mechanism through which **1d** inhibits cell growth, we analyzed the cell cycle profiles of multiple cell lines 24 hr after treatment with **1d** at different concentrations (Table 1). We found that **1d** treatment dramatically increased the number of cells in S-phase in H1299, HeLa, AGS and HepG2 cells at concentrations of 5 and 10 μg/ml, indicating S-phase cell cycle arrest (S1 Table and S2 Fig). U2OS cells were also arrested in S-phase by **1d** treatment but only in response to a lower concentration (2.5 μg/ml); at a higher concentration (7.5 μg/ml), **1d** lost this ability (S1 Table and S2 Fig). HCT116 cells were arrested in S-phase by **1d** at even lower concentrations (1 μg/ml and 2.5 μg/ml); at higher concentrations (5–10 μg/ml), a substantial percentage of the cells were shifted into the subG1 phase, which is indicative of apoptosis (S1 Table and S2 Fig). These results suggest that **1d** can induce S-phase cell cycle arrest in multiple cell lines, which showed different sensitivities and tolerance.

**Table 1:** Cell cycle distribution of human cancer cell lines after treatment for 24 hr with DMSO or 1d.

		Distribution (% cells)^a^			
Cell line	Treatment (μg/ml)	Sub-G1	G1	S	G2/M
H1299	DMSO	0	55.4	28.97	15.63
	5	0	30.97	69.03	0
	10	0	48.34	51.66	0
HepG2	DMSO	0	65.47	18.21	16.32
	5	0	33.92	66.02	0.06
	10	0	42.8	57.2	0
HeLa	DMSO	0	63.7	27.53	8.77
	5	0	45.4	52.97	1.63
	10	0	61.46	38.19	0.35
AGS	DMSO	0.79	50.8	18.07	31.12
	5	3.97	46.26	52.88	0.87
	10	12.44	49.03	50.79	0.19
U2OS	DMSO	0	60.44	25.42	14.14
	2.5	0	52.6	47.4	0
	7.5	0	66.28	21.17	12.55
HCT116	DMSO	3.59	43.05	40.11	16.84
	1	3.37	21.63	54.64	23.73
	2.5	8.31	23.34	73.61	3.05

^a^ DNA content was analyzed using PI staining and DNA flow cytometry. The data indicate the percentage of cells in each phase of the cell cycle. All experiments were conducted in duplicate and gave similar results.

### Compound 1d induces apoptosis in HCT116 cells

**Fig 2:**
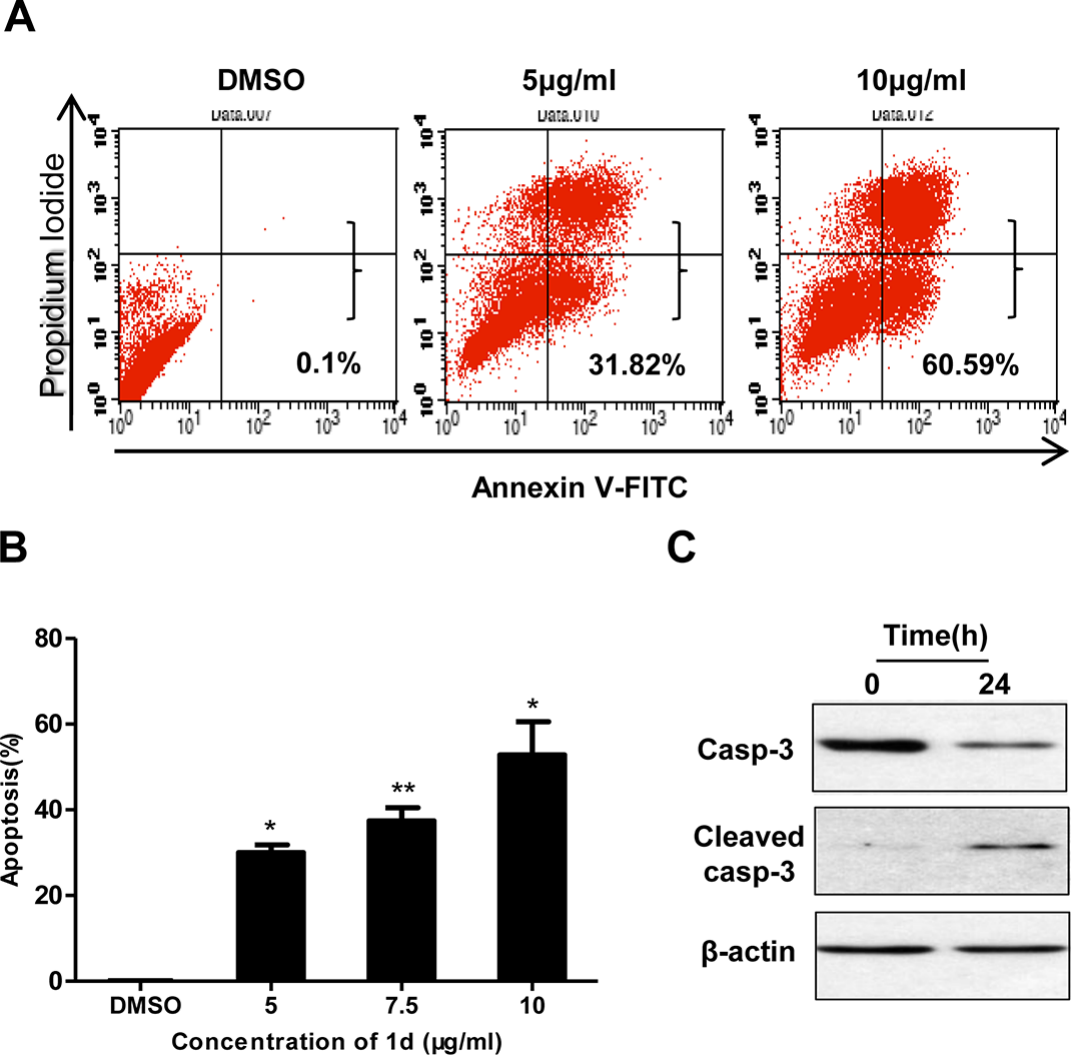
Apoptosis analysis induced by 1d in HCT116 cells. (A) Representative plots of flow cytometry analyses of HCT116 cells treated with **1d** or DMSO (control) for 24 h followed by Annexin V-FITC and PI staining. (B) The percentage of apoptotic cells in the above analysis. Data are the means of 2 independent experiments ± SEM. *, p<0.05; **, p<0.005. (C) HCT116 cells treated as above were analyzed using Western blotting with an antibody that recognizes both full length and cleaved caspase-3. β-actin was used as an internal loading control.

To confirm that **1d** indeed triggered apoptosis in HCT116 cells, we performed Annexin V and PI double staining followed by FACS analysis. As shown in Fig 2A and 2B and **1d** can induce apoptosis in HCT116 cells in a dose-dependent manner as the number of both early (Annexin V positive/PI negative) and late (Annexin V positive/PI positive) apoptotic cells are significantly increased. Accordingly, cleavage of caspase 3 was detected (Fig 2C). We also performed Annexin V and PI double staining for other cell lines, including HeLa, U2OS, H1299 and HepG2 cells, and we did not observe significant apoptosis in these cells (S2 Table). The sensitivity of AGS to **1d** was between the above groups and HCT116 cells (S2 Table). **1d** also did not induce apoptosis in IMR90 cells (S3 Fig). These results indicate that HCT116 is sensitive to **1d**-induced apoptosis.

### Compound 1d activates p53

**Fig 3:**
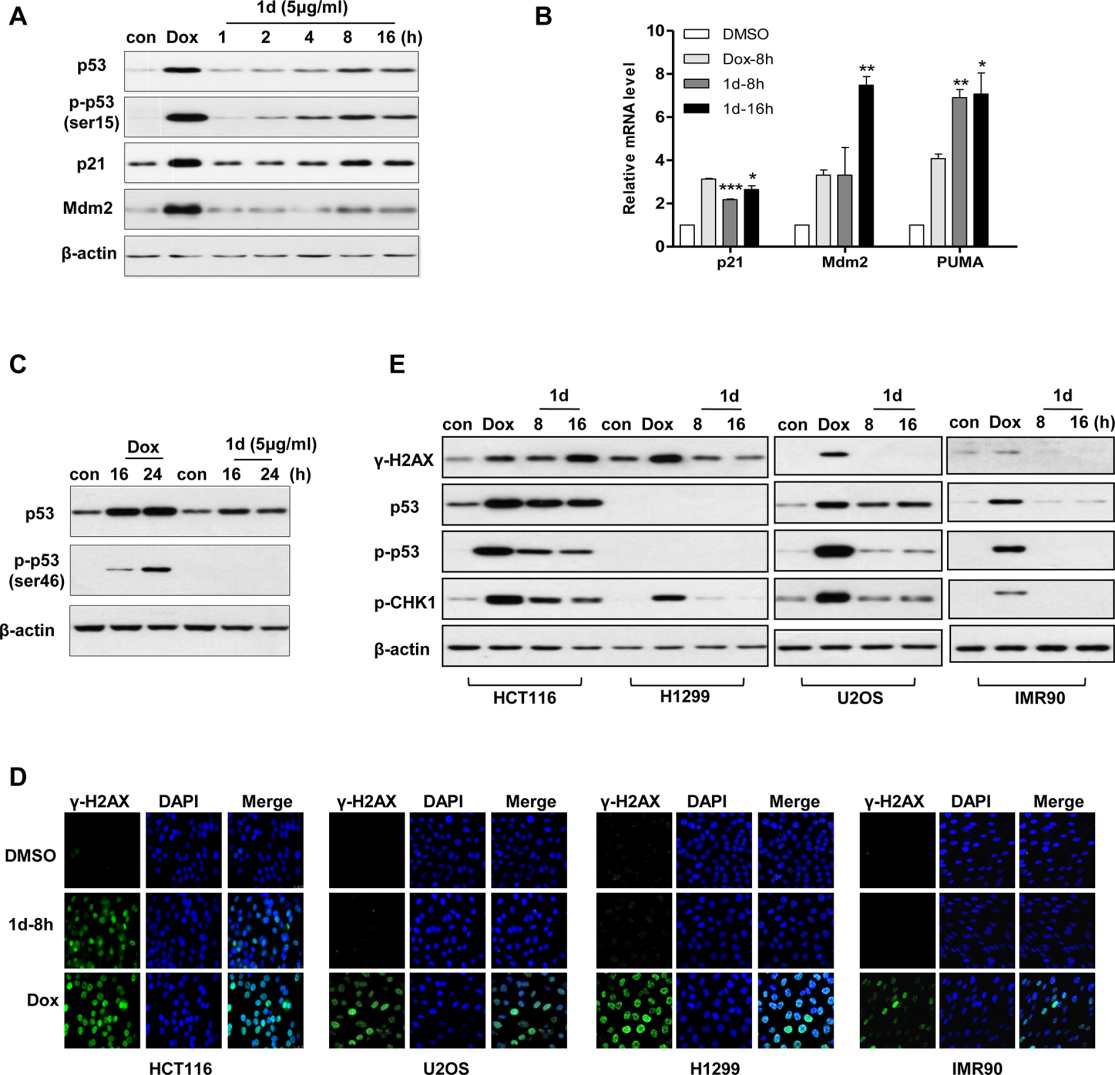
p53 activation and DNA damage is induced by 1d in HCT116 cells. (A-C) HCT116 cells treated with control (DMSO), Dox or **1d** were analyzed using Western blot using the indicated antibodies (A and C) or were subjected to real-time PCR analysis (B). Data are the means of 2 independent experiments ± SEM. *, p<0.05; **, p<0.005; ***, p<0.001. (D and E) HCT116, H1299, U2OS and IMR90 cells treated with DMSO, Dox or **1d** were immunostained followed by fluorescence microscopy (D) or were analyzed using Western blot using the indicated antibodies (E).

p53 activation has been implicated in apoptosis triggered by multiple types of stresses, as indicated by an increase in its protein levels and upregulation of its target genes. We therefore examined whether **1d** induced p53 activation in HCT116 cells. Western blot analysis showed that the protein levels of p53 as well as its target gene, p21, were upregulated upon **1d** treatment, suggesting that p53 is activated (Fig 3A). However, compared with the commonly used chemotherapeutic drug Dox the ability of **1d** to activate p53 is weaker. Nevertheless, the modestly increased p53 was transcriptionally competent as the p53 target genes, including p21, Mdm2 and PUMA, were transcriptionally increased (Fig 3A and 3B).

p53 modification is closely related to its activation [15]. We therefore examined whether **1d** induced p53 phosphorylation and found that p53 was phosphorylated on Ser15 (Fig 3A) but not on Ser46 (Fig 3C). p53 S15 phosphorylation is mainly mediated by ATM or CHK2, two prominent kinases in DNA damage response [16–20]. p53 S15 phosphorylation suggests that **1d** may trigger the DNA damage response in HCT116 cells. To address this possibility, we immuno-stained γ-H2A.X, a widely used marker for DNA double strand breaks [21, 22]. As shown in Fig 3D, a significant amount of γ-H2A.X was detected, suggesting that **1d** indeed induced DNA damage. Although Dox treatment could induce γ-H2A.X foci formation in U2OS, H1299 and IMR90 cells, **1d** failed to do so. Western analysis further confirmed the above observation (Fig 3E). Therefore, **1d** appeared to selectively induce DNA damage response in HCT116 cells.

### p53 is involved in 1d-induced apoptosis in HCT116 cells

**Fig 4:**
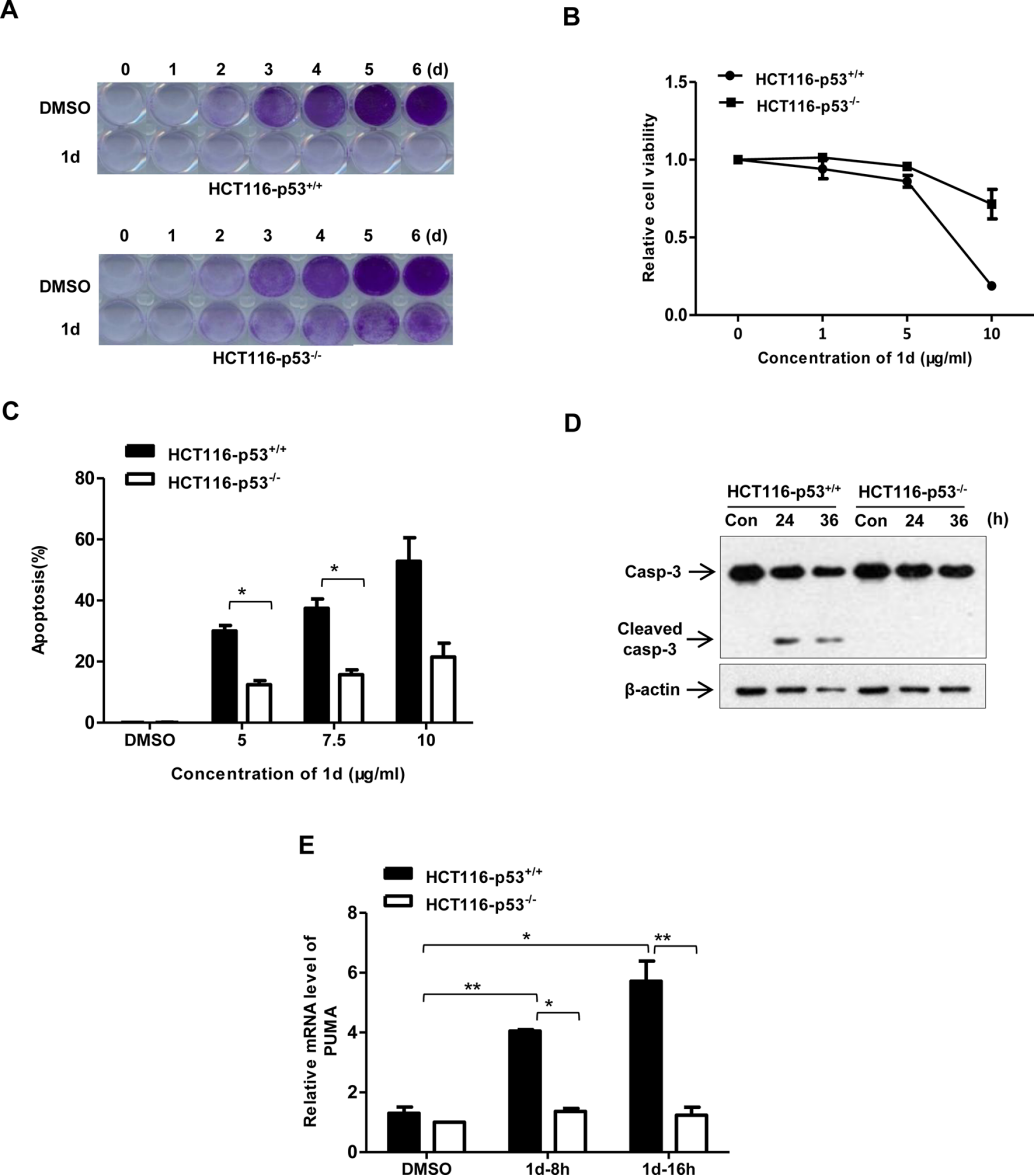
1d-induced apoptosis in HCT116 cells is partially dependent on p53. (A) HCT116 (p53^+/+^) and HCT116-p53^-/-^ cells were treated with 1 μg/ml of **1d** or DMSO (control) for the indicated time periods and then stained with crystal violet. (B) HCT116 cells were treated with **1d** at the indicated concentrations or DMSO for 48 h and then evaluated using the MTT assay. Data are the means of 2 independent experiments ± SEM. (C) The percentage of apoptotic cells in HCT116 cells treated with 5 μg/ml of **1d** or DMSO for 24 hr followed by flow cytometry analysis. Data are the means of 2 independent experiments ± SEM. *, p<0.05 when comparing HCT116 and HCT116-p53^-/-^ cells. (D) HCT116 cells were treated with 5 μg/ml of **1d** for the indicated durations and then analyzed using Western blot using a caspase 3 antibody. (E) The cells were treated with 5 μg/ml of **1d** and then subjected to real-time PCR analysis. Data are the means of 2 independent experiments ± SEM (n = 2). *, p<0.05; **, p<0.005 when comparing with vehicle controls or when comparing HCT116-p53^+/+^ and HCT116-p53^-/-^ cells.

We next examined whether p53 is involved in **1d**-induced apoptosis. We took advantage of a pair of HCT116 cells (p53^-/-^ and p53^+/+^) and compared their sensitivity to **1d** with respect to cell proliferation and apoptosis. Compared with that of p53 wild-type HCT116 cells, the cell growth inhibitory effect of **1d** on p53^-/-^ cells was markedly reduced (Fig 4A and 4B). Apoptosis analysis revealed that **1d**-induced apoptosis in p53 wild-type cells was much more prominent than that of p53^-/-^ cells (Fig 4C). Consistently, the kinetics of Caspase 3 cleavage was slower in p53^-/-^ cells (Fig 4D). Moreover, the **1d**-induced upregulation of PUMA, a target of p53 and a pro-apoptotic protein, in HCT116 cells was abrogated in p53^-/-^ cells (Fig 4E). Taken together, these data indicate that p53 is involved in **1d**-induced cell growth inhibition and apoptosis. However, **1d** still retains the ability to suppress the growth of p53-deficient cells, such as H1299 and HCT116-p53^-/-^, which suggests that other, unknown mechanisms also contribute to its anti-tumor activity.

### Compound 1d suppresses the growth of xenograft tumors *in vivo*

**Fig 5:**
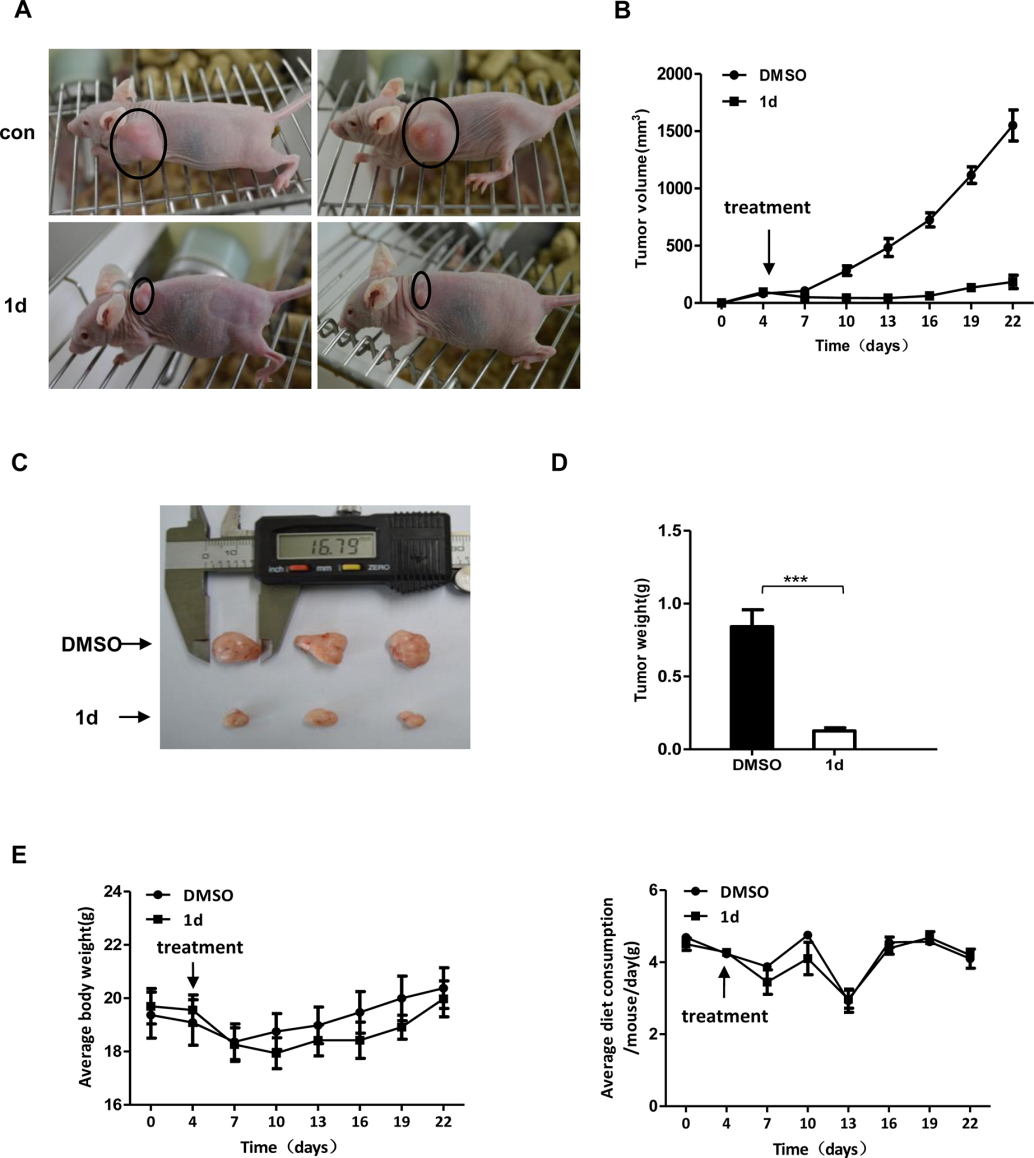
1d inhibits the growth of tumors derived from HCT116 cells. HCT116 cells were injected into athymic BALB/c nu/nu mice to induce tumors, and the mice were then treated with **1d** or DMSO. (A) Representative mice are shown at the end of the treatment. (B) Tumor volume was measured every 3 days after the first injection. (C) Images of representative tumors that were excised at the end of the treatment are shown. (D) The average tumor weights of the control versus the **1d**-treated group are depicted graphically. (E) Body weight and average diet consumption were recorded. Data are the means ± SEM (n = 6). ***, p<0.001, compared with the control.

**Fig 6:**
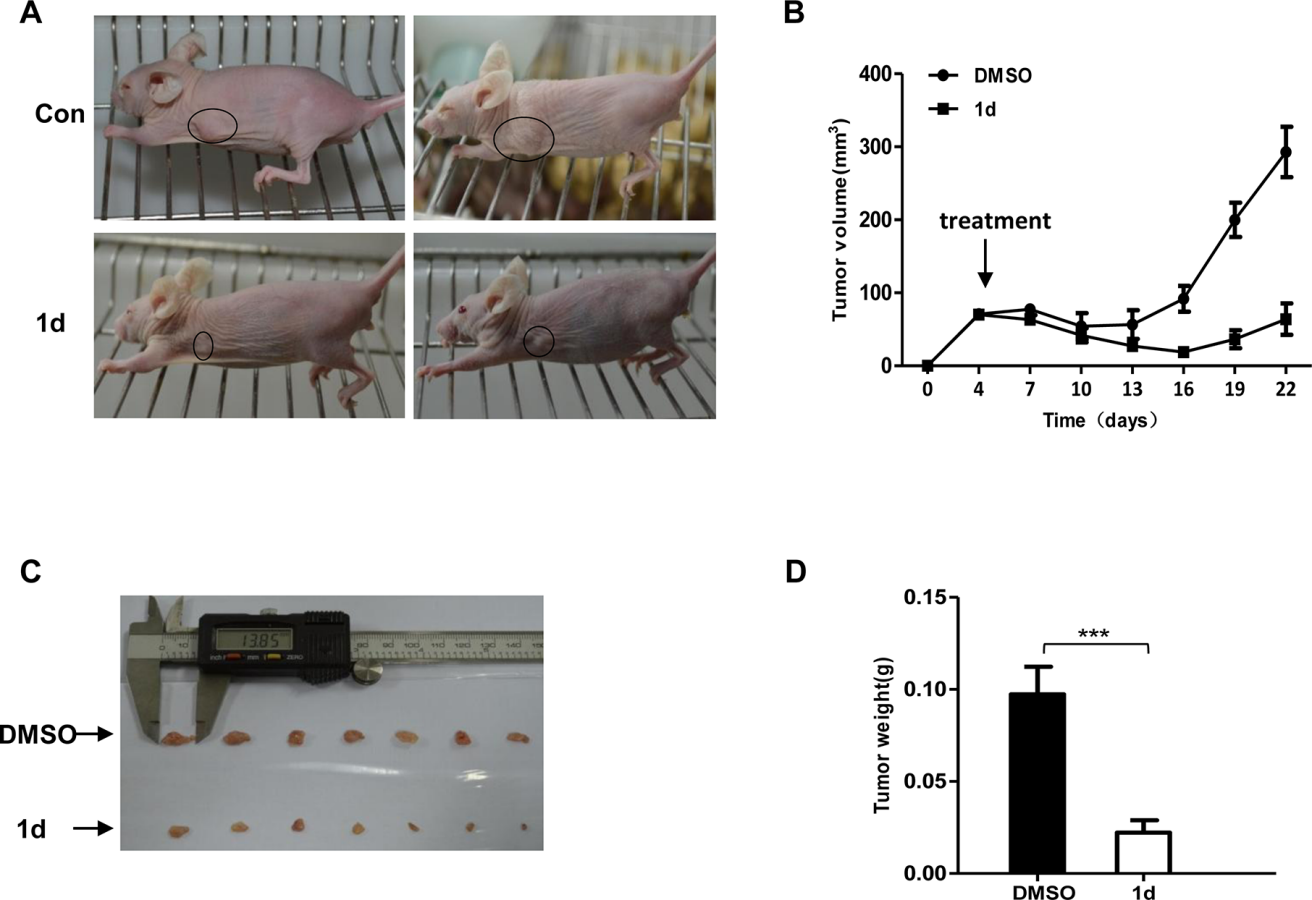
1d inhibits the growth of tumors derived from H1299 cells. H1299 cells were injected into athymic BALB/c nu/nu mice to induce tumors, and the mice were then treated with **1d** or DMSO. (A) Representative mice at the end of the treatment are shown. (B) Tumor volume was measured every 3 days after the first injection. (C) Images of all the excised tumors are shown. (D) The averaged tumor weights of the control versus the **1d**-treated group are depicted graphically. Data are the means ± SEM (n = 7). ***, p<0.001, compared with the control.

Next, we investigated the effect of **1d** on tumor growth in mouse models. To establish tumors, we implanted HCT116 or H1229 cells into the left flank of athymic nude mice. When tumors were palpable, **1d** treatment was started. Compared with vehicle-treated controls, **1d** treatment significantly reduced the growth of HCT116 (Fig 5A–5D) and H1299 (Fig 6) tumor xenografts. To check for side effects, we measured the body weights and diet consumption, and no significant difference was observed throughout the experiment (Fig 5E and 5F).

## Discussion

In this study, we explored the antitumor activity of the derivatives of 5-hydoxy-1*H*-pyrrol-2-(5*H*)-one and demonstrated that **1d** possessed a strong anti-proliferative activity toward a wide range of human cancer cell lines, and that the normal human IMR90 cells were relatively insensitive to **1d**-induced growth inhibition. **1d** induced S-phase cell cycle arrest and apoptosis. We further showed that **1d** efficiently suppressed the growth of HCT116 and H1299 cell-based xenograft tumors in *vivo*. Our results suggest that **1d** is an effective agent for cancer treatment.

Compound **1d** suppressed the growth of multiple cancer cell lines derived from various origins including colon, bone, lung, stomach, liver and the cervix. Based on the responses to **1d**, these cell lines can be categorized into three groups. The first group includes HeLa, H1299 and HepG2. These cells were arrested in S-phase by **1d** in response to a wide range of concentrations without undergoing apoptosis. However, permanent cell cycle arrest in S-phase would lead to apoptosis. The second group includes U2OS, which can be arrested in S-phase by **1d** only at low concentrations; high concentrations of **1d** could not induce this arrest. Thus, for this group of cells, low doses of **1d** activate the S-phase checkpoint, and this induction was abrogated by high doses of **1d**. The third group includes HCT116 and AGS. A striking feature for this group of cells, particularly HCT116 cells, is that these cells are sensitive to **1d**-induced apoptosis instead of cell cycle arrest. However, in the *in vivo* tumor models, **1d** effectively suppressed the proliferation of both HCT116 and H1299 tumors, suggesting that **1d**-induced S-phase cell cycle arrest is most likely the key mechanism for its anti-tumor activity *in vivo*.

The induction of S-phase arrest is a common response for different types of cells to **1d** treatment at cytostatic doses. Even for the apoptosis-sensitive cell line HCT116, cytostatic doses of **1d** could still induce S-phase arrest. S-phase progression is controlled by the binding of CDK2 to cyclin A or cyclin E, which is further activated by Cdc25A mediated dephosphorylation, therefore any perturbations that affect the complexes formation or activation would result in S-phase cell cycle arrest. Although S-phase checkpoint is often associated with DNA replication stress or DNA repair process, in which Cdc25A is phosphorylated and inactivated by ATR/CHK1 pathways, S-phase cell cycle arrest is also observed in many other scenarios through mechanisms affecting the expression of Cyclins or CDKs. For instance, Ashimori N et al showed that the small-molecule inhibitor of Bcl-2 TW-37 mediates S-phase cell cycle arrest and suppresses head and neck tumor angiogenesis [23]. Tong WG et al also showed that the leukotriene B4 receptor antagonist, LY293111 induces S-phase cell cycle arrest and reduces the expression of CDK2, cyclin A and cyclin E [24]. The observation that DNA damage marker γ-H2A.X was detected only in the apoptosis-sensitive cell line HCT116 but not in other cell lines indicated that **1d** induced S-phase cell cycle arrest is not due to the DNA damage response, but rather through other mechanisms that are commonly existed in all these cell lines. However, understanding these mechanisms require further investigation in depth.

An interesting observation in this study is that **1d** at a concentration close to the IC50 appeared to selectively induce apoptosis in HCT116 cells. We further demonstrated that **1d** specifically induced a DNA damage response in HCT116 cells and that p53 is involved in **1d**-induced apoptosis. The fact that the topoisomerase II inhibitor Doxorubicin induces γ-H2AX accumulation in a wide range of cell types and that **1d** failed to do so hints that **1d** may not directly target DNA but rather act through a pathway that is unique to HCT116 cells. Because HCT116 cells are defective in mismatch repair due to a lack of hMLH1 [10], we examined whether mismatch repair defects contributed to **1d**-induced apoptosis (S4 Fig). However, knockdown of hMLH1 in HeLa (S4B Fig) and another colon cancer cell line, HT29 (S4C Fig), did not sensitize these cells to **1d**-induced apoptosis, which suggests that the mismatch repair defect in HCT116 cells may not be responsible for its sensitivity to **1d**. We also explored whether ROS production contributed to **1d**-induced DNA damage in HCT116 cells because ROS induces DNA damage [25]. Although ROS was detected after treating the cells with **1d** for 24 hr, no substantial amounts of ROS were produced at the early time points, when γ-H2AX was already evident (S5 Fig). Thus, ROS is not a likely source for **1d**-induced DNA damage in HCT116 cells. It would be interesting to determine the molecular mechanism by which **1d** selectively induces apoptosis in HCT116 cells.

5-hydoxy-1*H*-pyrrol-2-(5*H*)-one is an important N-heterocyclic scaffold that occurs in both natural products and medicinal chemistry. Our results showed that all the derivatives we synthesized exhibited some degrees of anti-proliferative activity, suggesting that both the N-heterocyclic scaffold and the side groups contribute to this activity. More importantly, we demonstrated that **1d** possesses a potent tumor suppression activity using both *in vitro* cell culture and *in vivo* xenograft tumor models, suggesting the potential use of **1d** as an anti-tumor agent.

## Supporting Information

S1 Dataset (DOCX)

S1 Fig Chemical structures of the derivatives of 5-hydoxy-1*H*-pyrrol-2-(5*H*)-one. (TIF)

S2 Fig Cell cycle analysis in different cell types treated with 1d. The cells indicated were treated with **1d** at the indicated concentrations for 24 h, followed by cell cycle analysis using flow cytometry.
(TIF)

S3 Fig Apoptosis analysis in HCT116, U2OS and IMR90 cells treated with 1d. The cells indicated were treated with 5 μg/ml of **1d** for 24 and 48 h, and then subject to PI staining followed by flow cytometry analysis. Sub-G1 population was considered as apoptotic cells.
(TIF)

S4 Fig hMLH1 is not involved in 1d-induced apoptosis in HCT116 cells. A, HCT116 and HT29 cells were treated with **1d** or DMSO (control) for 24 h and then stained with Annexin V-FITC and propidium iodide, followed by flow cytometry analysis. The percentages of apoptotic cells shown in the right panel. Data are shown as the means of 2 independent experiments ± SEM. The effect of hMLH1 knockdown on **1d**-induced apoptosis in HeLa (B) and HT29 cells (C). The cells were transfected with hMLH1 and non-silencing siRNA for 48 h and then treated with **1d** (5 μg/ml) or DMSO (control) for 24 h. The knockdown efficiency of hMLH1 was analyzed using Western blot, as shown on the top panels. Apoptosis was analyzed and is shown in the lower panels.
(TIF)

S5 Fig ROS production measurement in HCT116 cells treated with 1d. The cells were treated with **1d** (5 μg/ml) or DMSO (control) for 1, 3, 6, or 24 h, and then the levels of ROS were detected as described in S1 Dataset.
(TIF)

S1 Table IC50s of the compounds that inhibit the growth of HCT116 cells. (DOCX)

S2 Table Apoptosis induction in human cancer cell lines after treatment with DMSO or 1d for 24 h. (DOCX)
