# Supplementary material for: Antitumor Activity of a 5-Hydroxy-1H-Pyrrol-2-(5H)-One-Based Synthetic Small Molecule In Vitro and In Vivo
Source: PLoS One. 2015 Jun 4;10(6):e0128928. doi: 10.1371/journal.pone.0128928 (PMC4456381; doi:10.1371/journal.pone.0128928)
Supplement: S1 Dataset — (DOCX) [file pone.0128928.s001.docx]

**S1 Dataset**

**Quantitative RT-PCR**

Total RNA was isolated as described in Materials and Methods. The same amounts of cDNA products were amplified by PCR. The following primers were used: hMLH1: 5’-gctgatgttaggacactacc-3’and 3’-gttgattctaccagacgatgg-5’;

**siRNA and transfection**

siRNAs were transfected into HeLa and HT29 cells using RNAiMAX (Invitrogen), respectively, following the manufacturer's instructions. The target sequences for non-silencing and hMLH1were described in (1) and (2) respectively.

**Detection of reactive oxygen species (ROS) level**

Intracellular ROS generation was assessed using the stable nonpolar dye 2,7 -dichlorofluorescein-diacetate (DCFH-DA) that readily diffuses into cells. HCT116 cells were treated with **1d** for 1, 3, 6 or 24 h in the presence or absence of NAC or PDTC, and then incubated with 10 μM of DCFH-DA for 30 min followed by washing twice with cold PBS. ROS production was measured by flow cytometry using a flow cytometer.

**References**

1. Li L, Cui D, Zheng SJ, Lou H, Tang J. Regulation of Actinomycin D induced upregulation of Mdm2 in H1299 cells. DNA repair 2012;11(2):112-9.

2. Mastrocola AS, Heinen CD. Nuclear reorganization of DNA mismatch repair proteins in response to DNA damage. DNA repair 2010;9(2):120-33.
