# Supplementary figures and images for: Antitumor Activity of a 5-Hydroxy-1H-Pyrrol-2-(5H)-One-Based Synthetic Small Molecule In Vitro and In Vivo
Source: PLoS One. 2015 Jun 4;10(6):e0128928. doi: 10.1371/journal.pone.0128928 (PMC4456381; doi:10.1371/journal.pone.0128928)

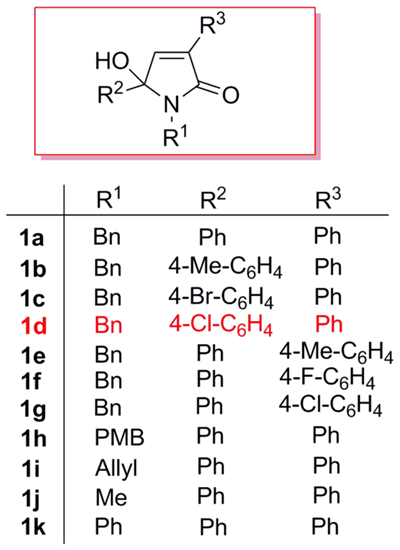

Supplement: S1 Fig — (TIF) [file pone.0128928.s002.tif]

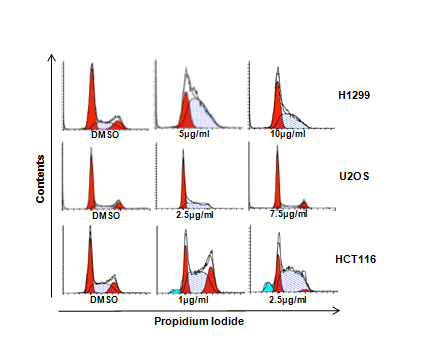

Supplement: S2 Fig — The cells indicated were treated with 1d at the indicated concentrations for 24 h, followed by cell cycle analysis using flow cytometry. (TIF) [file pone.0128928.s003.tif]

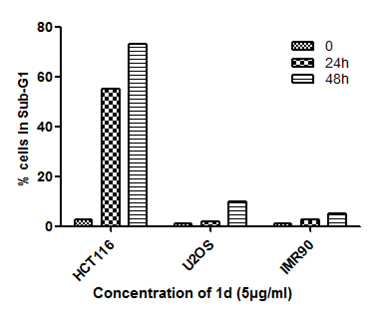

Supplement: S3 Fig — The cells indicated were treated with 5 μg/ml of 1d for 24 and 48 h, and then subject to PI staining followed by flow cytometry analysis. Sub-G1 population was considered as apoptotic cells. (TIF) [file pone.0128928.s004.tif]

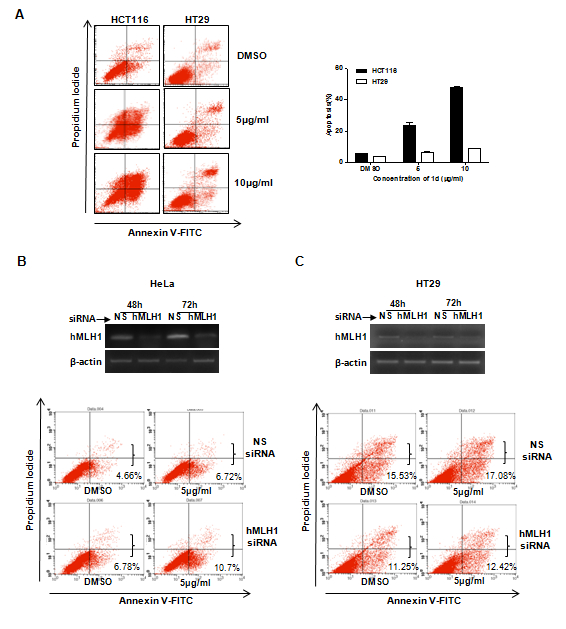

Supplement: S4 Fig — A, HCT116 and HT29 cells were treated with 1d or DMSO (control) for 24 h and then stained with Annexin V-FITC and propidium iodide, followed by flow cytometry analysis. The percentages of apoptotic cells shown in the right panel. Data are shown as the means of 2 independent experiments ± SEM. The effect of hMLH1 knockdown on 1d-induced apoptosis in HeLa (B) and HT29 cells (C). The cells were transfected with hMLH1 and non-silencing siRNA for 48 h and then treated with 1d (5 μg/ml) or DMSO (control) for 24 h. The knockdown efficiency of hMLH1 was analyzed using Western blot, as shown on the top panels. Apoptosis was analyzed and is shown in the lower panels. (TIF) [file pone.0128928.s005.tif]

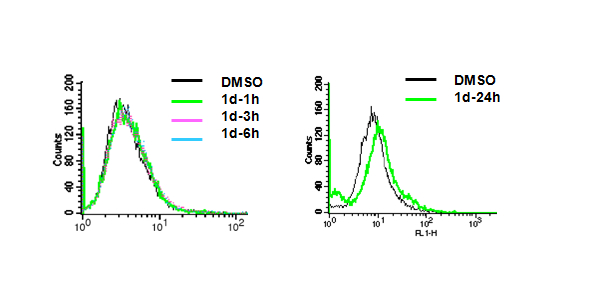

Supplement: S5 Fig — The cells were treated with 1d (5 μg/ml) or DMSO (control) for 1, 3, 6, or 24 h, and then the levels of ROS were detected as described in S1 Dataset. (TIF) [file pone.0128928.s006.tif]
