# Supplementary material for: Antitumor Activity of a 5-Hydroxy-1H-Pyrrol-2-(5H)-One-Based Synthetic Small Molecule In Vitro and In Vivo
Source: PLoS One. 2015 Jun 4;10(6):e0128928. doi: 10.1371/journal.pone.0128928 (PMC4456381; doi:10.1371/journal.pone.0128928)
Supplement: S1 Table — (DOCX) [file pone.0128928.s007.docx]

**S1 Table**  IC50s of the compounds inhibiting the growth of HCT116 cells

| Compound ^a^ | IC50 ^b^(μM) |
| --- | --- |
| 1a | 47 |
| 1b | 27 |
| 1c | >100 |
| **1d** | **13** |
| 1e | 25 |
| 1f | 43 |
| 1g | 23 |
| 1h | 67 |
| 1i | >100 |
| 1j | >100 |
| 1k | 26 |
| Dox | 2.6 |

^a^ Chemical structures of the compounds were shown in Supplementary Fig. 1. ^b^ IC50s of the compounds were determined using MTT assay after treating HCT116 cells for 48 h. All the experiments were conducted in duplicates and gave similar results
