# Supplementary material for: Antitumor Activity of a 5-Hydroxy-1H-Pyrrol-2-(5H)-One-Based Synthetic Small Molecule In Vitro and In Vivo
Source: PLoS One. 2015 Jun 4;10(6):e0128928. doi: 10.1371/journal.pone.0128928 (PMC4456381; doi:10.1371/journal.pone.0128928)
Supplement: S2 Table — (DOCX) [file pone.0128928.s008.docx]

**S2 Table** Apoptosis induction in human cancer cell lines after treatment for 24h with DMSO or **1d**

|  | % Apoptosis ^a^ | | |
| --- | --- | --- | --- |
| Cell line | DMSO | 5μg/ml | 10μg/ml |
| HCT116 | 0.1 | 31.82 | 60.59 |
| AGS | 2.12 | 16.45 | 36.62 |
| H1299 | 3.21 | 3.2 | 2.83 |
| U2OS | 2.71 | 7.01 | 9.69 |
| HepG2 | 1.68 | 3.78 | 3.82 |
| HeLa | 0.74 | 4.7 | 15.34 |

^a^ Apoptosis was determined using an Annexin V-based assay (Fig. 2). The data indicate the percentage of apoptotic cells in each sample. All the experiments were conducted in duplicates and gave similar results.
